# Langerhans Cell Histiocytosis Manifests with Acute Severe Hypernatremia during Hospitalization

**DOI:** 10.1155/2022/6120644

**Published:** 2022-10-14

**Authors:** Kullaya Takkavatakarn, Hansamon Poparn, Pisut Katavetin

**Affiliations:** ^1^Division of Nephrology, Department of Medicine, King Chulalongkorn Memorial Hospital, Faculty of Medicine, Chulalongkorn University, Bangkok, Thailand; ^2^Department of Pediatric Hematology and Oncology, Department of Pediatrics, Faculty of Medicine, Chulalongkorn University, Bangkok, Thailand; ^3^Integrative and Innovative Hematology/Oncology Research Unit, Faculty of Medicine, Chulalongkorn University, Bangkok, Thailand

## Abstract

Central diabetes insipidus (DI) is characterized by a deficiency in arginine vasopressin (AVP), an antidiuretic hormone leading to excessive free water loss in the urine and hypernatremia. Central DI can be the first presentation of several occult diseases. However, patients with central DI who have functioning thirst mechanisms and access to water may initially exhibit normal sodium levels. We report a 57-year-old woman who was admitted to the hospital due to cholangitis. Her initial serum sodium was normal and she rapidly developed severe hypernatremia after fluid restriction. The results of the laboratory workup indicated DI, which dramatically responded to desmopressin. MRI showed an ill-defined faint hyper signal intensity in T1, T2/FLAIR lesions involving the bilateral hypothalamus. The histopathological findings confirmed the diagnosis of Langerhans cell histiocytosis (LCH) with multiorgan involvement. Serum sodium returned to normal after receiving desmopressin and water replacement therapy.

## 1. Introduction

Hypernatremia, defined as a serum sodium concentration greater than 145 mEq/L, is a common electrolyte disorder in hospitalized patients and is significantly associated with in-hospital mortality. The primary cause of hyponatremia is water deficit resulting from a deficiency in water intake or excessive water loss. Central diabetes insipidus (DI) is characterized by a lack of arginine vasopressin (AVP), an antidiuretic hormone (ADH), due to several disorders that arise from either the pituitary or the hypothalamus. The deficiency of AVP leads to hypotonic polyuria. Central DI patients who have intact thirst mechanisms increase their water intake to maintain water balance and sodium levels. In the setting of impaired or limited access to free water, hypernatremia might rapidly develop. Delay in diagnosis and treatment of central DI could result in severe hypernatremia and serious neurological complications. Central DI should be considered in patients who develop acute hypernatremia after hospitalization, especially in patients with polyuria, regardless of the history of hypothalamic-pituitary disease.

## 2. Case Report

A 57-year-old woman was admitted to the medical ward due to an acute fever for 2 days. She had palpable neck mass and progressive jaundice for 2 months. She developed nocturia and polydipsia with a daily fluid intake of 4–6 L per day for 2 weeks. On physical examination, her blood pressure was 82/50 mmHg, pulse rate 92 beats per minute, and body temperature 38°C. Her body weight was 58 kg. She had moderate icteric sclera and thyroid enlargement with a firm consistency. Her liver span was 14 cm. Her abdomen was soft with mild tenderness at the right upper quadrant. The computed tomography of the neck and abdomen revealed diffusely enlarged thyroid glands that caused tracheal effacement and marked hepatomegaly with many enlarged intra-abdominal lymph nodes that constricted the common bile duct. She was diagnosed with acute cholangitis with septic shock. Her vital signs were stable after receiving antibiotics and resuscitation with 2 L of normal saline. She was ordered to be NPO to monitor her abdominal signs. Her serum sodium was 138 mEq/L. Eighteen hours after admission, her serum sodium level increased to 169 mEq/L without overt neurological abnormalities. Total fluid intake was 3,800 ml (normal saline) and her urine output was 5,850 mL in 18 hours. Her body weight was 56.4 kg.

**Figure 1:**
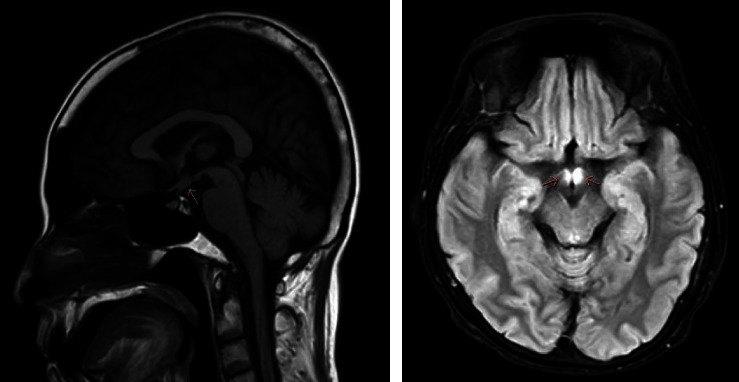
T1 and T2 FLAIR sections of magnetic resonance imaging show hyper signal intensity involving the bilateral hypothalamus.

**Figure 2:**
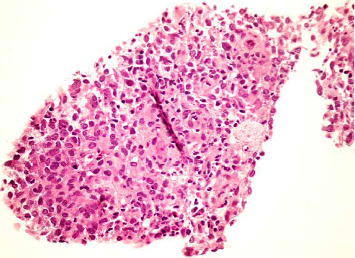
The core biopsy shows the aggregation of histiocyte-like cells, some of which show nuclear grooves. Mitosis and necrosis are not observed.

Routine laboratory values are shown in Table 1. Urine specific gravity and osmolality were 1.004 and 152 mOsm/Kg, respectively. Urine sodium was less than 20 mEq/L and urine potassium was 6.8 mEq/L. After two micrograms of desmopressin was prescribed, her urine output was substantially reduced to 20 mL per hour and urine osmolality was increased to 628 mOsm/Kg (313%), indicating complete central DI. The hormonal assay revealed normal serum morning cortisol and thyroid hormone. Magnetic resonance imaging (MRI) of the sellar and suprasellar regions was performed to determine the underlying pathology responsible for central DI. The pituitary gland and pituitary stalk were both normal in size. There was an ill-defined faint hyper signal intensity in T1, T2/FLAIR lesion involving the bilateral hypothalamus with modest perilesional edema (Figure 1). The pathology of the thyroid and lymph node biopsies revealed aggregation of histiocyte-like cells, some of which had nuclear grooves. The immunohistochemistry was positive for CD1a and S100, which was compatible with Langerhans cell histiocytosis (LCH) (Figure 2). She had no family history of LCH. Her bone and skin were unremarkable.

**Figure 3:**
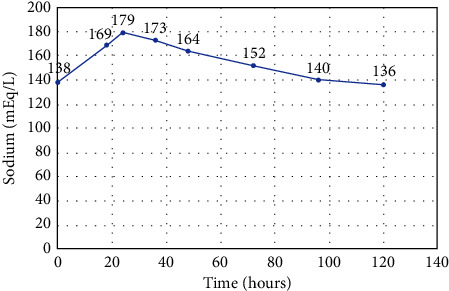
Time course of sodium levels.

The final diagnosis was complete central DI due to LCH with the hypothalamus, thyroid, and lymph node involvement. To correct hypernatremia, 5% dextrose water was administered to replace the water deficit. In addition, daily intranasal desmopressin 2.5 mcg was prescribed. Her serum sodium level had returned to normal after three days.  Figure 3 demonstrates the time course of the serum sodium concentration.

After treatment with intravenous antibiotics, acute cholangitis was significantly improved and the patient could be discharged from the hospital. Dexamethasone was started for treatment of LCH and chemotherapy was scheduled to begin. Two weeks later, the patient was still stable, with a normal serum sodium level.

## 3. Discussion

Hypernatremia is a common electrolyte disorder caused by either excessive sodium intake or net water loss. Only a few instances, such as administration of hypertonic sodium bicarbonate during resuscitation or hypertonic saline to correct hyponatremia, cause sodium retention. Therefore, the cause of hypernatremia in the vast majority of patients is water deficit due to decreased water intake or increased water loss. Thirst and ADH secretion are the two defense mechanisms against hypernatremia. Although stimulation of thirst occurs at a slightly higher serum osmolality than ADH secretion, thirst is the primary defense mechanism against hypernatremia. Thus, patients with normal thirst mechanisms and who can access water rarely have hypernatremia. In addition, ADH plays an essential role in preventing further electrolyte-free water loss by maximizing urine concentration [1]. Three primary conditions are necessary to concentrate urine: (1) a concentrated renal medullary interstitium, (2) ADH to insert aquaporin-2 channels into the apical membranes of the collecting duct, and (3) the ability of collecting duct cells to respond to ADH [2]. Patients with defective thirst mechanisms or limited access to water, such as neonates, the elderly, and patients with neurological disabilities, may develop hypernatremia due to insufficient water intake. DI and osmotic diuresis result in excess urinary water loss. While diarrhea, vomiting, and conditions that enhance insensible loss cause nonrenal water loss.

**Figure 4:**
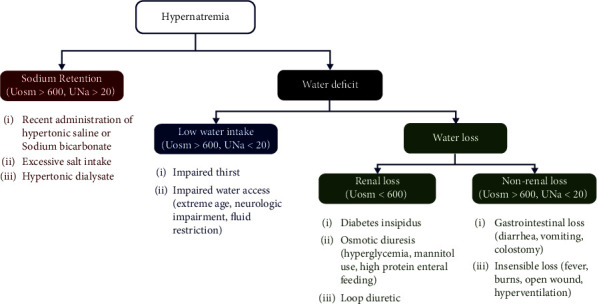
Diagnostic approach to hypernatremia.

Hypernatremia is always accompanied by hyperosmolarity. As a result, patients with hypernatremia should have maximally concentrated urine. If defense mechanisms are intact and the renal concentrating mechanism is normal, patients would have urine osmolality greater than 600–800 mOsm/Kg and would not have polyuria [3]. A normal response is observed in patients with hypodipsia and nonrenal water loss. In contrast, patients with lower urine osmolality or polyuria usually have hypernatremia from diabetes insipidus or osmotic diuresis. In our patient, her serum sodium levels increased significantly (31 mEq/L in 18 hours) after being admitted to the hospital with oral fluid restriction. The patient did not have a history of hypertonic fluid administration and gastrointestinal loss. Moreover, the patient had low urine osmolality and polyuria (>3 L in 24 hours), suggesting renal water loss from diabetes insipidus (Figure 4).

The next step diagnostic process was to determine whether the DI is central or nephrogenic. Administration of desmopressin, a synthetic analog of ADH, after water deprivation, could help distinguish the various forms of DI. Patients with central DI are able to concentrate the urine after the deficiency of ADH is replaced with desmopressin, whereas patients with nephrogenic DI do not have a substantial response to desmopressin due to renal resistance to ADH activity. An increase of at least more than 50 percent in urine osmolality after desmopressin administration suggests complete central DI which is caused by several disorders that arise from either the pituitary or the hypothalamus [4]. A thorough clinical history and physical examination should be obtained to evaluate for the signs and symptoms of hormonal deficiency. Also, an MRI of the sellar and suprasellar regions is required to evaluate any pituitary or hypothalamic abnormalities.

LCH is a rare disorder characterized by clonal proliferation of CD1a+/CD207+ myeloid dendritic cells. The pathogenesis of LCH remains unclear, but recent studies support that activation of the mitogen-activated protein kinase (MAPK) pathway plays a major role in the development of LCH, with somatic mutation in BRAF^V600E^ (50–65%) and MAP2K1 (25%) being the most common mutations resulting in the overactive MAPK pathway [5, 6]. LCH is primarily considered a pediatric disease with a case rate of approximately 5 per million; however, it can occur at any age. The incidence of LCH in an adult is very low (1–2 cases per million) [7]. Consequently, the diagnosis of LCH might be delayed due to the lack of suspicion.

The clinical manifestations of LCH depend on sites of accumulation or infiltration of abnormal proliferative cells, ranging from focal self-limited single organ involvement to aggressive multiorgan involvement. Bone and skin are the most common sites, followed by the central nervous system (CNS), liver, spleen, lungs, lymph nodes, and hematopoietic system [8]. Adult-onset LCH is typically multisystem involvement. The most common presenting symptom of CNS-LCH is central DI due to hypothalamic-pituitary involvement [9]. The prevalence of central DI in patients with LCH ranges from 10–30% and increases to 40% in patients with multisystem involvement [10, 11]. A previous case of adipsic DI caused by LCH has been previously reported. The infiltration of the thirst center in the hypothalamus could be the possible mechanism underlying adipsic DI [12]. In addition, CDI can be the first manifestation, even before LCH is diagnosed. Therefore, LCH should be considered in patients with central DI. MRI brain may show abnormal T2 hyper-intensity at hypothalamic or sella-suprasellar regions. The definitive diagnosis of LCH must be confirmed by a pathological study of affected tissue that should be positive for CD1a, CD207, and S100 in immunohistochemical studies [13].

## 4. Learning Points

- Central DI patients with intact thirst mechanisms and water accessibility can present with normal sodium levels. A detailed history of polyuria and polydipsia should be obtained in suspicious cases.
- Central DI should be considered in acute severe hypernatremia patients with polyuria regardless of the history of hypothalamic-pituitary disease.
- Langerhans cell histiocytosis, an uncommon illness that affects multiple organs, is one of the causes of central DI. Therefore, a biopsy of the affected tissue should be performed to confirm the diagnosis when the clinical picture is suspicious of this disease.

## Figures and Tables

**Table 1:** Laboratory data.

Parameter	Result	Reference
*Serum*		
Sodium, mEq/L	138	135–145
Potassium, mEq/L	3.4	3.5–5.0
Chloride, mEq/L	102	98–107
Bicarbonate, mEq/L	31	22–29
Urea nitrogen, mg/dL	9	7–20
Creatinine, mg/dL	0.78	0.5–1.0
Glucose, mg/dL	98	70–100
Calcium, mg/dL	7.7	8.8–10.2
Phosphorus, mg/dL	2.8	2.5–4.5
Albumin, g/dL	1.8	3.5–5.0
Total bilirubin, mg/dL	11.26	0.2–1.2
Direct bilirubin, mg/dL	8.65	0–0.5
SGOT, *μ*/l	121	5–35
SGPT, *μ*/l	21	0–40
Alkaline phosphatase, *μ*/L	900	40–120

*Urine*		
Specific gravity	1.004	1.003–1.030
Osmolality, mOsm/Kg	152	100–850
Sodium, mEq/L	<20	
Potassium, mEq/L	6.8	

SGOT-serum glutamic-oxaloacetic transaminase; SGPT-serum glutamic pyruvate transaminase.

## Data Availability

The data that support the findings of this study are available from the corresponding author, upon reasonable request.
